# *Pedilanthus tithymaloides* Inhibits HSV Infection by Modulating NF-κB Signaling

**DOI:** 10.1371/journal.pone.0139338

**Published:** 2015-09-25

**Authors:** Durbadal Ojha, Rashmi Das, Parveen Sobia, Vedprakash Dwivedi, Soma Ghosh, Amalesh Samanta, Debprasad Chattopadhyay

**Affiliations:** 1 ICMR Virus Unit, ID and BG Hospital, General Block 4, 57 Dr Suresh Chandra Banerjee Road, Beliaghata, Kolkata 700010, India; 2 Department of Microbiology, College of Cell Sciences, University of KwaZulu-Natal, Durban 4001, South Africa; 3 Department of Pharmaceutical Technology, Jadavpur University, Raja SC Mallick Road, Kolkata 700032, India; National Center for Cell Science, INDIA

## Abstract

*Pedilanthus tithymaloides* (PT), a widely used ethnomedicinal plant, has been employed to treat a number of skin conditions. To extend its utility and to fully exploit its medicinal potential, we have evaluated the *in vitro* antiviral activity of a methanolic extract of PT leaves and its isolated compounds against Herpes Simplex Virus type 2 (HSV-2). Bioactivity-guided studies revealed that the extract and one of its constituents, luteolin, had potent antiviral activity against wild-type and clinical isolates of HSV-2 (EC_50_ 48.5–52.6 and 22.4–27.5 μg/ml, respectively), with nearly complete inhibition at 86.5–101.8 and 40.2–49.6 μg/ml, respectively. The inhibitory effect was significant (p<0.001) when the drug was added 2 h prior to infection, and was effective up to 4 h post-infection. As viral replication requires NF-κB activation, we examined whether the observed extract-induced inhibition of HSV-2 was related to NF-κB inhibition. Interestingly, we observed that treatment of HSV-2-infected cells with extract or luteolin suppressed NF-κB activation. Although NF-κB, JNK and MAPK activation was compromised during HSV replication, neither the extract nor luteolin affected HSV-2-induced JNK1/2 and MAPK activation. Moreover, the PT leaf extract and luteolin potently down-regulated the expression of tumor necrosis factor (TNF)-α, Interleukin (IL)-1β, IL-6, NO and iNOS and the production of gamma interferon (IFN-γ), which are directly involved in controlling the NF-κB signaling pathway. Thus, our results indicate that both PT leaf extract and luteolin modulate the NF-κB signaling pathway, resulting in the inhibition of HSV-2 replication.

## Introduction

Diseases caused by Herpes Simplex Virus (HSV), includes mild cold sores, keratoconjunctivitis, genital herpes, and life-threatening encephalitis, affect more than one-third of the global population. Following its entry through bruised skin or mucosal surfaces, HSV primarily infects epithelial cells and is finally transported to the nerve ganglia for life-long latency with periodic reactivation [1]. After entry, HSV triggers innate immune responses that eventually lead to antigen-specific adaptive immune responses, but the virus is able to overcome these responses to establish latent infection. HSV infection is known to require enhanced activation of NF-κB [2–4] and MAPK in targeted epithelial and nerve cells [5–9], and compound(s) that block these pathways can inhibit HSV infection. Resveratrol, a natural stilbenoid, and curcumin, a diarylheptanoid, were reported to inhibit HSV infection through NF-κB inhibition [10, 11]. NF-κB, MAPKs and JNK transcriptionally control the production of most cytokines and chemokines [5, 12, 13].

*Pedilanthus tithymaloides* L. Poit. (Euphorbiaceae) or devil's-backbone is a low tropical shrub, locally known as Rang-chita. Different parts of the plant are used in traditional Indian medicine as abortive, anticancer, anti-inflammatory and antimicrobial [14–16], with therapeutic activity against skin disorders [17]. We previously isolated the flavonoid luteolin along with the new compound tetradecanediol sodium salt from the methanolic extract (ME) of PT leaves [15]. Luteolin has been reported to suppress NF-κB activation and COX-2 expression triggered by the MyD88-independent pathway via Toll-like receptor-4 [18]. It has also been reported that luteolin inhibits the post-attachment stage of Enterovirus 71 and Coxsackie virus A16 [19], replication of Poliovirus [20], Influenza virus A [21], and HIV-1 [22]. Further, crude extracts from *Avicenna marina* inhibit HSV plaque formation [23], wheras *Houttuynia cordata* extracts inhibit HSV infection through inhibition of NF-κB activation [24]. Furthermore, treatment with 7,3'-disulfate luteolin or luteolin from *Zostera marina* induced increased survival of mice infected with the tick-borne encephalitis [25]. However, in depth studies on the antiviral activity and mechanisms of action, including the regulatory mechanism of PT leaves against HSV-2 have not yet been reported. Following ethnomedicinal use, we evaluated the *in vitro* anti-HSV-2 activity of ME from PT leaves (MEPT) and its isolated compound(s) in a bioactivity-guided study, and investigated their possible mechanisms of action. We observed that MEPT and luteolin exhibited potent anti-HSV-2 activity, blocked HSV-2 infection through the inhibition of NF-κB activation, but had no effect on virus-induced JNK and MAPK activation.

## Material and Methods

### Materials

RPMI 1640, penicillin and streptomycin were from Sigma Chemical Co. (St Louis, MO, USA); Dulbecco modified Eagle medium (DMEM) and fetal calf serum (FCS) were from Gibco BRL (Grand Island, NY, USA). ELISA antibody kits (IL-1α, IL-6, IFN-γ and TNF-α) were purchased from BD Biosciences (San Diego, CA); while Santa Cruz Biotechnology (Santa Cruz, CA) and Abcam^®^ (Cambridge, MA, USA) supplied antibodies against IκB-α, NF-κB/p65, MAPK/p38, p-p38, JKN1/2, p-JNK1/2 and GAPDH. Tissue culture reagents were purchased from Gibco Laboratories, USA; and primers for semi-quantitative PCR were from IDT, California, USA. Other chemicals were purchased from Sigma (USA) or Merck (Germany).

### Plants

The PT leaves was collected from the Begumpur village of Baruipur Block, 24-Parganas (S), West Bengal and were identified by a taxonomist at the Indian Botanic Garden, Botanical Survey of India, Howrah, India. The voucher specimen (CNH/1-1(56)/2006/Tech-11/1450) has been deposited at the host institute.

### Extraction and bioactivity-guided isolation

The extraction protocols were similar as described earlier [14, 15], and the bioactivity-guided isolation has been described in **S1 File**.

### Cells and viruses

Vero cells (ATCC, USA) were cultured in DMEM and RPMI-1640, respectively, with 5% FCS, penicillin (100 U/mL) and streptomycin (100 μg/ml) at 37°C in 5% CO_2_. The viral strains include HSV-2G (ATCC734), two clinical isolates (ICMR/VU-2012-17, 20) and a TK-deficient HSV-2 isolate (ICMR/VU-2013-7) provided by Professor P.K. Dutta, Department of Dermatology, Calcutta Medical College & Hospital, Kolkata. All virus strains were passaged in Vero cells.

### Preparation of mouse peritoneal macrophages

The animals used were female and male BALB/c mice (18–20 gm), acclimatized for 15 days in the Animal House facility, with standard food and water *ad libitum*. The animal experiments were conducted in accordance with the OECD guidelines, and as approved by the Institutional Animal Care and Use Committee (IACUC) of Jadavpur University, Kolkata (Approval No: 0367/01/C/CPCSEA). Seven-week-old mice were intraperitoneally injected with 1 ml of 4% thioglycolate. After 5 days the animals were subjected to Ketamine hydrochloride (100 mg/kg i.m.) anesthesia to minimize suffering, and euthanized by cervical dislocation. The peritoneal cells were harvested by ice-cold PBS, and centrifuged at 1200 rpm in 4°C for 5 min. The cell pellet was suspended in RPMI-1640 supplemented with 10% FCS, and the cells were counted on Neubaur’s chamber. The cells were cultured for 6 h at 37°C in 5% CO_2_, washed with PBS to remove the non-adherent cells, and further incubated for 24 h [26].

### Cytotoxicity and antiviral assay

The Vero cells treated with serially diluted MEPT, isolated compounds luteolin and tetradecanediol, were incubated at 37°C in 5% CO_2_, using acyclovir (ACV) and DMSO (0.1%) as controls. After 72h, MTT assay was carried out using commercial kit, following the manufacturer’s protocol (MTT; Sigma) and absorbance was read at 570 nm. The 50% cytotoxic concentration (CC_50_) was calculated by linear regression of the dose-dependent curves while antiviral activity was measured by plaque reduction assay (PRA). Briefly, Vero cells infected with clinical isolates (VU/12-17, VU/2012/20 and VU/2013-7) or wild-type HSV-2G (100 PFU) were treated with serial dilutions of test drugs and then overlaid with 1% methylcellulose. The plaques were counted after 72 h to calculate the virus titers by scoring the plaque-forming units (PFU). The effective concentration of test drugs that reduced plaque numbers by 50% (EC_50_) was interpolated from the dose-response curves [27, 28].

### Time-of-addition assay

The effect of drug addition over time was carried out to determine the possible step(s) of viral life cycle targeted by MEPT or luteolin. Following three different approaches Vero cells were exposed to MEPT (86.5 μg/ml) or luteolin (40.2 μg/ml) before, during or after infection with HSV-2 (100 PFU). For pre-infection, cells were treated with MEPT or luteolin for 2 h, washed with PBS and then infected with HSV-2. In case of co-infection the cells were simultaneously exposed to HSV-2 and MEPT or luteolin, and after 1 h the virus-drug mixture was removed to conduct PRA of the treated cells. For post-infection (p.i) studies the cells were first infected with HSV-2 for 1 h, washed with PBS, and then treated with MEPT or luteolin at intervals of 4, 8, 12 and 24 h, and finally the cells were harvested after 24 h for PRA [28].

### Western blot analysis

The HSV-2G (10 m.o.i.) infected Vero cells were treated with MEPT (86.5 μg/ml) or luteolin (40.2 μg/ml) and after 4 h, equal amounts of protein (40 μg/sample) extract from whole cells were harvested in buffer (200 μl/well) containing 50nM Tris-Cl, 150mM NaCl, 1% NP-40, 1% Triton X-100, and 1% protease inhibitor cocktail. The soluble fraction was then separated by centrifugation (16000 g for 10 min) at 4°C, subjected to SDS-PAGE and blotted to pre-equilibrated PVDF membrane (Thermo Scientific, USA). The membrane was then blocked in 5% NFDM in 1X TBST (20 mM Tris, pH 7.5, 150 mM NaCl, 0.5% Tween 20), rinsed and incubated with specific antibody in 5% BSA at 4°C overnight. Immunoblotting was performed with peroxidase-labelled specific antibodies and visualized by ECL Western blot detection kit (Millipore, USA) [29, 30].

### Isolation of RNA and Semi-quantitative RT- PCR

RNA was isolated from the HSV-2G (5 m.o.i.) infected, MEPT (86.5 μg/ml) or luteolin (40.2 μg/ml) treated (12h p.i.) Vero cell/macrophages using RNeasy Mini kit (Qiagen, Germany). The total RNA in RNase-free water was mixed in 20 μl of RT mix (containing 5X VILO Reaction Mix, 10X SuperScript Enzyme Mix and DEPC treated water) and subjected to cDNA synthesis using the GeneAmp PCR System 9600 (Bio-Rad MJ Mini, Hercules, CA, USA). The cDNA (10%) was subjected to standard PCR amplification using the primers for iNOS genes, using *gapdh* as internal standard. The sequences of the forward and reverse primer are listed in Table 1. The respective DNA and proteins bands were analysed using a model GS-700 Imaging Densitometer and Molecular Analyst software (version 1.5; Bio-Rad Laboratories, CA, USA).

**Table 1:** Primers used in Real time-PCR assays.

Gene	Primer Sequence
ICP0	5’-GATCGGATCCGGCGCTGGGGAGAGACGAGAAACC-3’
	5’-GATCGTCGACCCGAGTGTTAGCTCCCCCTACTCC-3’
DNA pol	5’-CAGAACTTCTACAACCCCCA-3’
	5’-TAGATGATGCGCATGGAGTA-3’
iNOS	5’-CCCTTCCGAAGTTTCTGGCAGCAGC- 3’
	5’-GGCTGTCAGAGCCTCGTGGCTTTGG- 3’
GAPDH	5’-CAAGGCTGTGGGCAAGGTCA-3’
	5’-AGGTGGA AGAGTGGGAGTTGCTG-3’

### Measurement of Nitric Oxide and Cytokine release

Macrophages cultured in 24 well plates (1 × 10^6^ cells/well) were treated with HSV-2 (5 m.o.i.), and incubated for 1h. The activated cells were then treated with MEPT (100 μg/ml) or luteolin (20 μg/ml) for 24 h. The concentrations of NO and cytokines in the supernatant were determined by Griess reagent (Sigma, USA) [31] and by sandwich ELISA [26, 31], respectively.

### Statistical analysis

Results were expressed as SEM (*n* = 6) and the statistical analyses were performed with one-way analysis of variance (ANOVA), followed by Dunnett’s test. A value of *p* < 0.05 was considered to be statistically significant, compared with the respective control.

## Results

### Isolation and identification of anti-HSV compounds

**Fig 1:**
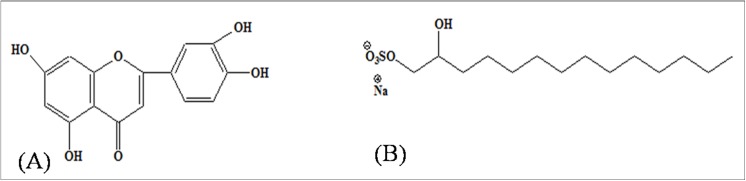
Chemical structure of the isolated compound(s) Luteolin (A) and Tetradecanediol (B) isolated from the antiviral fraction of *Pedilanthus tithymaloides* leave extracts.

The MEPT was partitioned between *n*-Butanol and water, and saturated with n-Butanol. The n-Butanol fraction separately chromatographed over a silica gel column resulted in the isolation of a flavone as compound-1 (**Fig 1A**), identified as 2-(3,4-Dihydroxy-phenyl)-5,7-dihydroxy-chromen-4-one or luteolin [14]; and a new tetradecanediol, sodium salt as compound-2 (**Fig 1B**), confirmed by comparing data in the literature [32].

### Inhibition of HSV-1 infection

The antiviral activity of MEPT leaves, isolated luteolin and tetradecanediol was determined against HSV-2G, two clinical isolates, and one TK-deficient stain of HSV-2 (100 PFU) using PRA in the presence of acyclovir and DMSO (0.1%) as control. The results showed a dose-dependent inhibition of viral plaque formation by both MEPT leaves and luteolin (data not shown) but tetradecanediol had no anti-HSV activity (**Table A in S1 File**). While the PRA demonstrated that MEPT leaves at 48.5–52.6 μg/ml and luteolin at 22.4–27.5 μg/ml inhibited all the strains tested, which was far below their CC_50_ concentration (**Table 2**). Further, nearly complete (EC_99_) inhibition of viral growth was achieved at 86.5 μg/ml of MEPT leaves or 40.2 μg/ml of luteolin against HSV-2G.

**Table 2:** Assessment of cytotoxicity and ant-HSV activity of MEPT leaves and Luteolin.

Virus	MEPT leaves			Luteolin			Acyclovir		
	CC_50_ ^a^	EC_50_ ^b^	SI^c^	CC_50_ ^a^	EC_50_ ^b^	SI^c^	CC_50_ ^a^	EC_50_ ^b^	SI^c^
HSV-2G	436.5±2.8	48.5±1.24	9.00	278.6±3.5	22.4±2.56	12.43	128.8±3.4	2.6 ± 0.8	49.53
HSV-2 CI 1	436.5±2.8	49.2±1.88	8.87	278.6±3.5	26.8±1.66	10.39	128.8±3.4	2.8 ± 0.3	46.00
HSV-2 CI 2	436.5±2.8	52.6±1.48	8.29	278.6±3.5	23.8±1.44	11.70	128.8±3.4	2.6 ± 0.6	49.53
TK^-^ strain	436.5±2.8	50.8±1.33	8.59	278.6±3.5	27.5±1.76	10.13	128.8±3.4	>30	-

CI, Clinical Isolates 1 and 2; TK^-^ Strain, Thymidine Kinase deficient strain

^a^ CC_50_, 50% cytotoxic concentration for Vero cells in μg/ml

^b^EC_50_, Concentration (μg/ml) producing 50% inhibition of virus-induced plaques in three separate experiments

^c^SI, Selectivity index (CC_50_/EC_50_).

### Effect of luteolin treatment at different times prior or after infection

**Fig 2:**
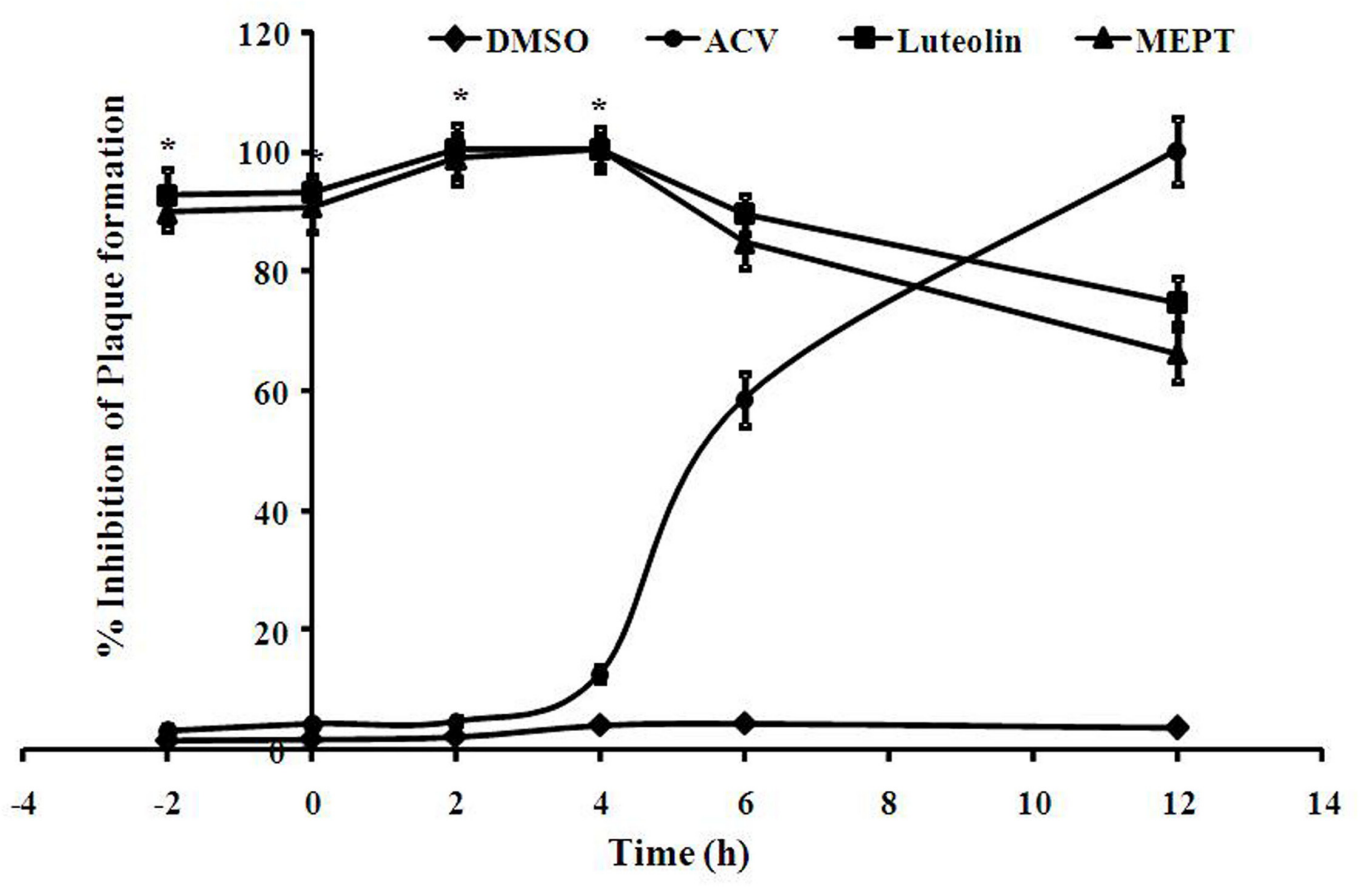
Inhibitory effect of MEPT leaves or isolated Luteolin in time-of-addition assays. Inhibitory effect of MEPT leaves, Luteolin or ACV (F) at various time points: pre-infection (-2 h), co-infection (0 h) and post-infection (1–12 h) with HSV-2G (100 PFU/well), determined by a plaque reduction assay. Each bar represents the mean ± S.E.M of three independent experiments (*, P<0.001, compare with control).

To identify the possible phase of viral infection imparted by MEPT leaves or luteolin, we performed time-of-addition assay. The results revealed that the inhibitory effect was significant when MEPT leaves or luteolin were added 2 h prior, during or 4h p.i., which correlates with the entry and early stages of virus infection (**Fig 2**).

### Effects of MEPT leaves or luteolin on HSV-2-induces NF-κB, MAPK, JNK1/2 and IκBα activation

**Fig 3:**
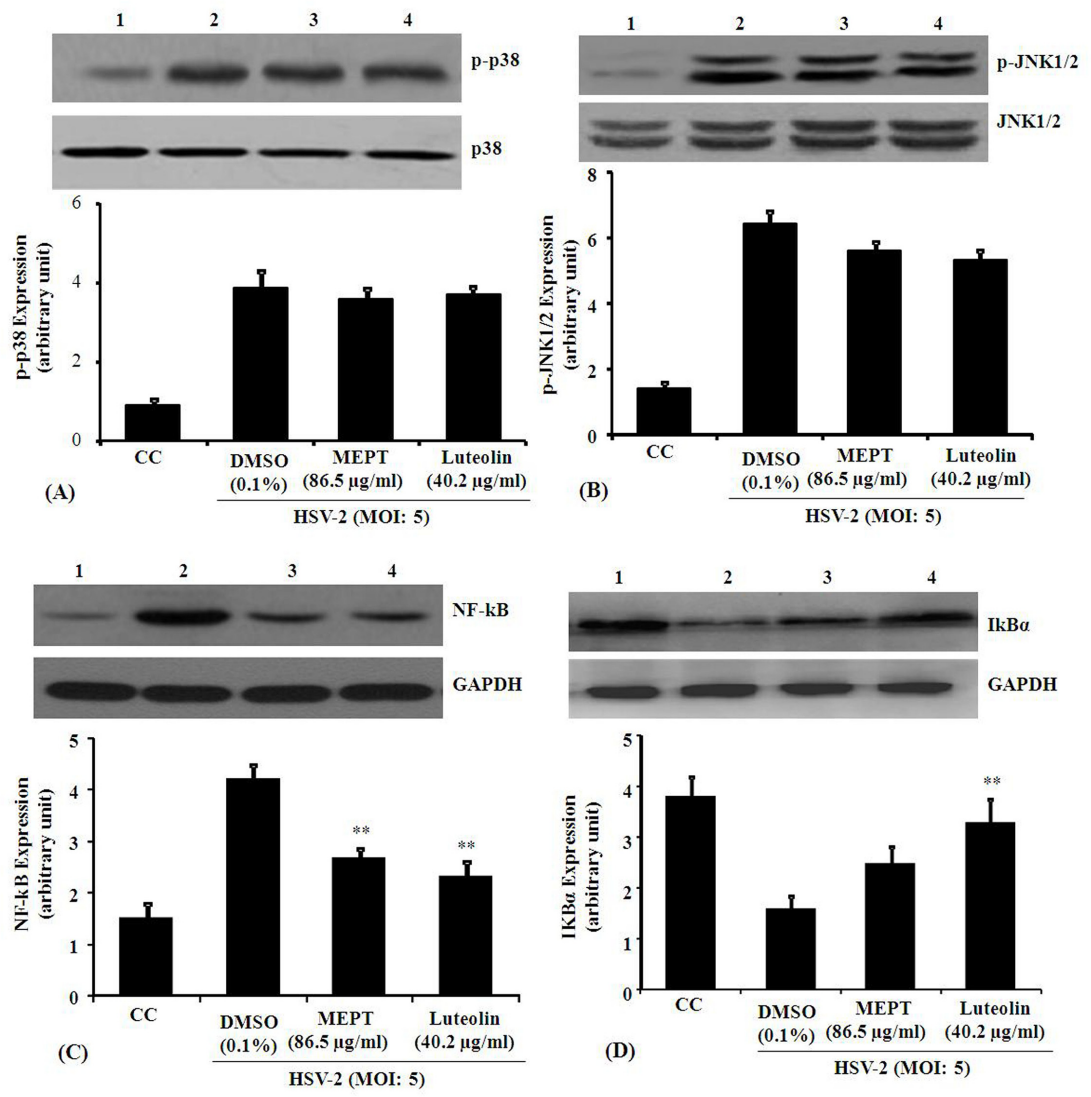
Effect of MEPT leaves and Luteolin on HSV-2-induced MAPK, JNK1/2, NF-kB and IkBα activation. Expression of MAPK (A), JNK (B), NF-kB (C) and IkBα (D) was determined by Western blot, using GAPDH as the internal control. The HSV-2-infected peritoneal macrophage(s) were treated with MEPT (86.5 μg/ml) or Luteolin (40.2 μg/ml) and after 4 h, equal amounts of protein (40 μg/sample) extract from whole cells were harvested in buffer (200 μl/well) containing 50 nM Tris-Cl, 150 mM NaCl, 1% NP-40, 1% Triton X-100, and 1% protease inhibitor cocktail. The soluble fraction was separated by centrifugation, subjected to SDS-PAGE and blotted to pre-equilibrated PVDF membrane. The membrane was then blocked in 5% NFDM in 1X TBST, rinsed and incubated with specific antibody at 4°C overnight. Immunoblotting was performed with peroxidase-labelled specific antibodies and visualized by ECL Western blot detection kit. The average expression of NF-kB was significantly higher in the HSV-2-induced macrophage, as compared to the control and MEPT or luteolin co-treated group (*, P<0.05; **, P<0.001). Lane 1, cells only control; Lane 2, cells + HSV-2; Lane 3, cells + HSV-2 + MEPT leaves; Lane 4, cells + HSV-2 + luteolin.

**Fig 4:**
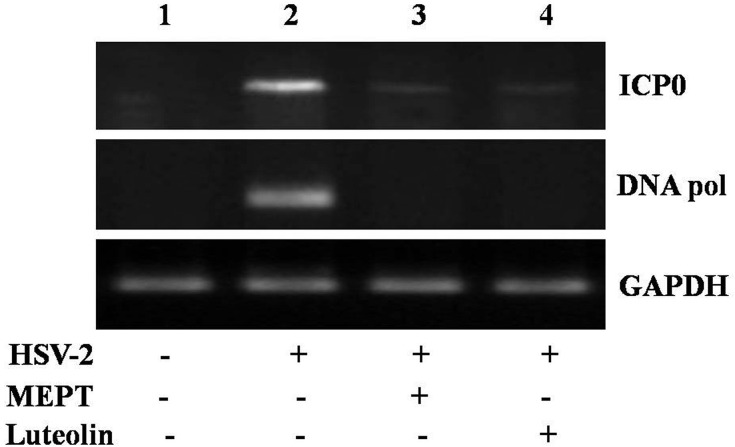
Effect of MEPT and Luteolin on HSV-2-induced ICP0 and DNA polymerase gene expression. The HSV-2 infected Vero cells were treated with the MEPT leaves or Luteolin, incubated for 12 h, following which RNA was isolated and subjected to RT–PCR analysis for expression of ICP0 and DNA pol mRNA. Lane 1, cells only control; lane 2, cells +HSV-2; Lane 3, cells + HSV-2 + MEPT leaves; Lane 4, cells + HSV-2 + luteolin.

As efficient HSV-2 replication requires NF-κB activation [3], and HSV-infection selectively promotes MAPK phosphorylation [5, 33], we examined whether MEPT leaves or luteolin block NF-κB, MAPK and JNK1/2 pathways during infection. HSV-2 infected cells, untreated or treated with MEPT leaves (86.5 μg/ml) or luteolin (40.2 μg/ml) for 4 h, were used to determine NF-κB nuclear translocation by immunoblotting. In uninfected cells, p65 was detected at relatively low levels, whereas, in HSV-2-infected cells p65 activation was significantly induced at 24 h p.i. MEPT leaves or luteolin treatment abated HSV-2-induced p65 nuclear translocation, suggesting that MEPT and luteolin blocked HSV-2-induced NF-κB activation, but not MAPK phosphorylation (**Fig 3**). As shown in **Fig 4**, MEPT or luteolin treatment significantly blocked viral ICP0 and ICP27 expression, implying inhibition of HSV-2 replication. Inhibition of IκB-α degradation induced following HSV-2 infection, was also detected in these samples. Together, our data suggest that MEPT/luteolin block HSV-2 infection through NF-κB inhibition.

### Luteolin reduces HSV-2-induced cytokine and NO production

**Fig 5:**
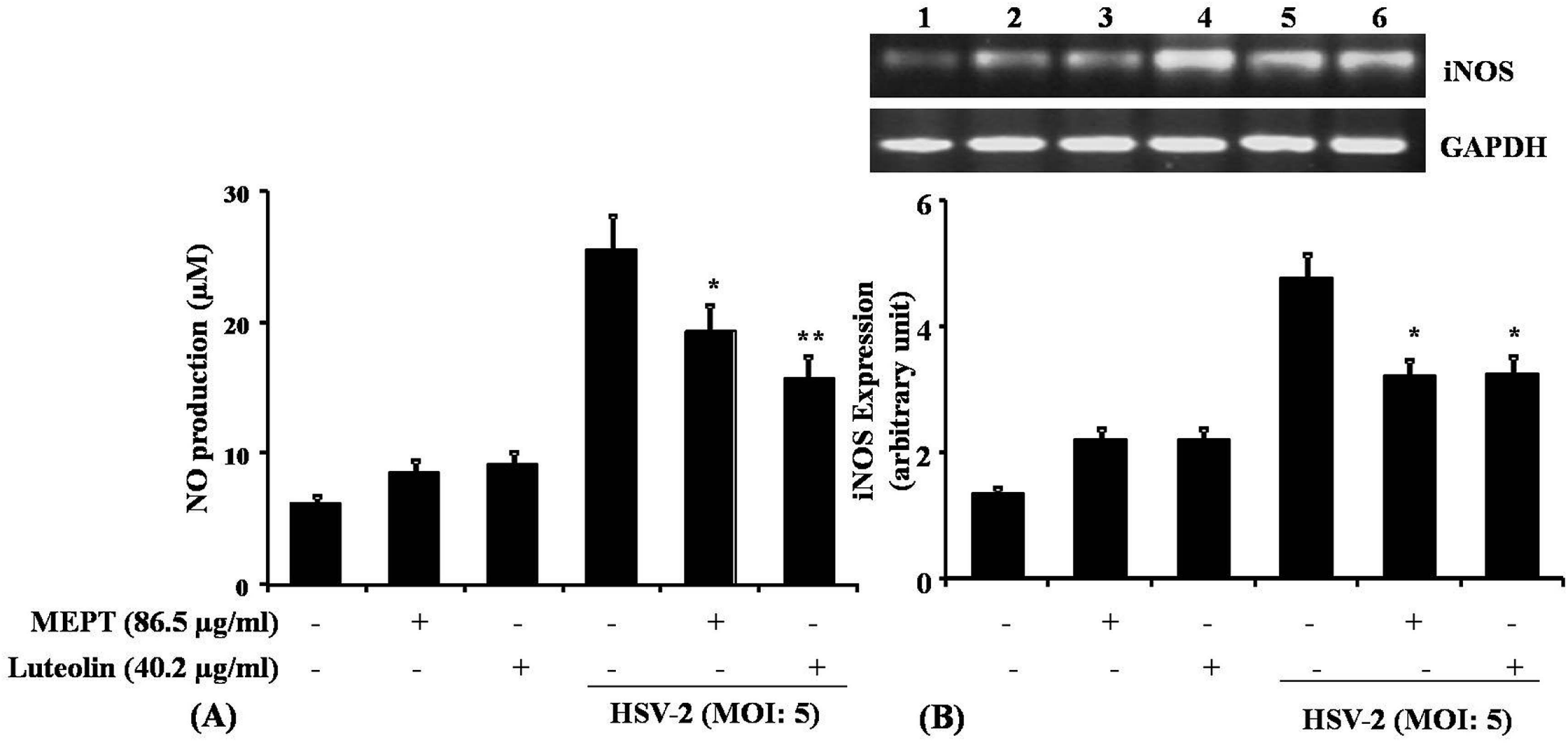
Effect of MEPT leaves and Luteolin on NO production and iNOS expression in HSV-2 infected murine macrophages. (A) Macrophages (10^6^ cells/ml) were infected with HSV-2 treated with the MEPT leaves (100 μg/ml) or Luteolin (20 μg/ml), and incubated for 24 h. The supernatant was removed and the concentration of NO was determined by Griess reagent. Data are expressed as Mean ± SD from triplicate experiments, yielding similar results (m moles of nitrite). Asterisks indicate a statistically significant increase (*, P<0.05; **, P<0.001) in nitrite generation, compared to the infected macrophages. (B) HSV-2-infected macrophages were treated with the MEPT leaves or Luteolin, incubated for 12 h, following which RNA was isolated and subjected to RT–PCR analysis for the expression of iNOS2 mRNA. The data are expressed as Mean ± SD from triplicate experiments yielding similar results. The asterisk indicates a statistically significant increase (*, P<0.001) in iNOS2 expression, compared to the infected macrophage.

**Fig 6:**
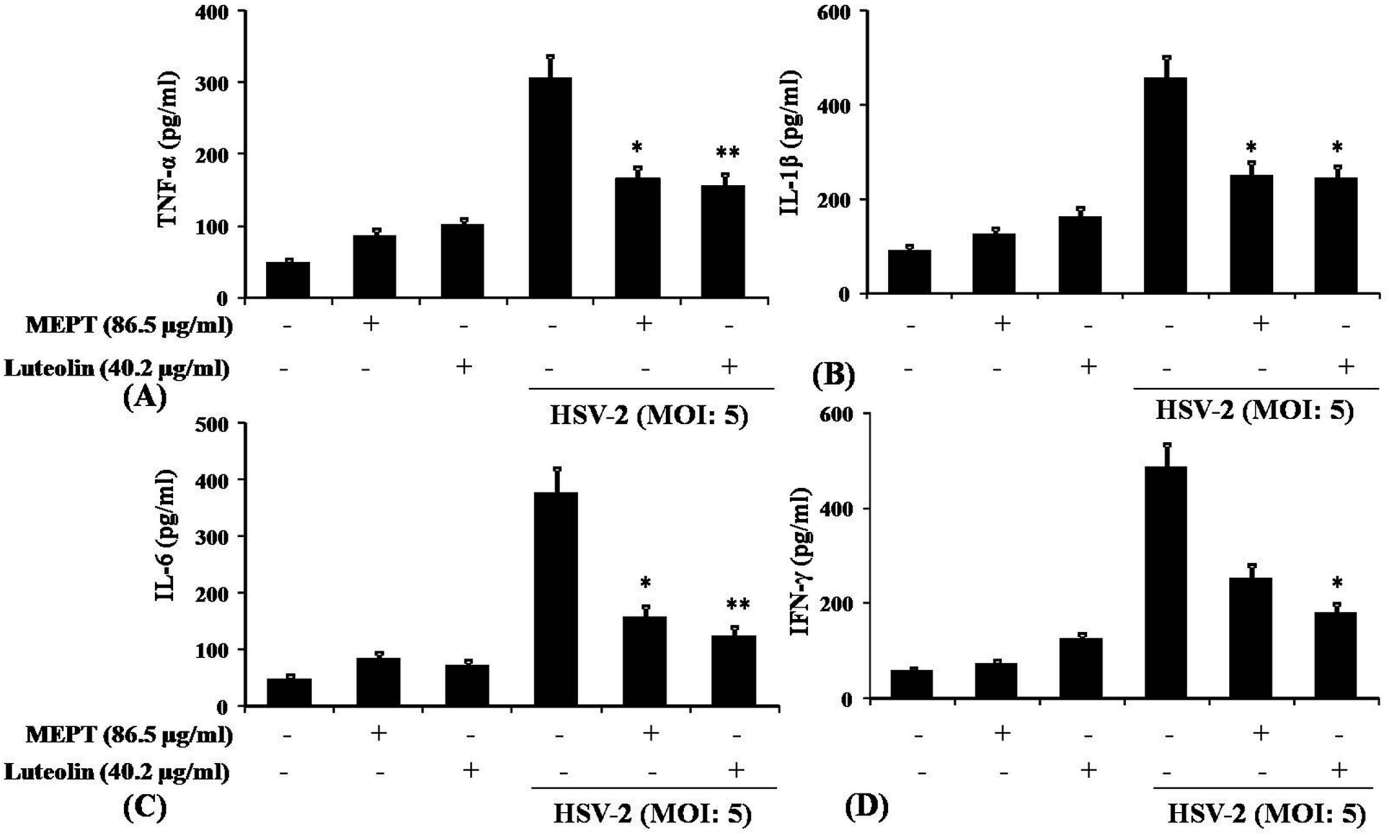
Effect of MEPT leaves and Luteolin on pro-inflammatory cytokine release in HSV-2-infected peritoneal macrophages. Peritoneal macrophages were cultured overnight and infected with HSV-2, washed after 1 h and then treated with MEPT leaves (100 μg/ml) or Luteolin (20 μg/ml). The cells were further incubated for 24 h, and the cell-free supernatants were subjected to sandwich ELISA to determine the level of (A) TNF-α, (C) IL-1β, (E) IL-6 and (G) IFN-γ (pg/mL). The ELISA data are expressed as Mean ± SD from triplicate experiments, yielding similar results. Asterisks indicate statistically significant (*, P<0.05; **, P<0.001) induction of TNF-α, IL-1ß, IL-6, and IFN-γ release, compared to the infected macrophages.

Because NF-κB regulates the production of many inflammatory cytokines during HSV-infection [26], we examined HSV-2-induced cytokines, NO and iNOS gene expression in mouse peritoneal macrophages treated with MEPT leaves or luteolin (**Fig 5**). We observed a significant down-regulation of TNF-α, IL-1β, IL-6 and IFN-γ, compared with the infection control, by ELISA and qPCR (**Fig 6**). NO and iNOS expression were suppressed in MEPT- or luteolin-treated HSV-2 infected macrophage, compared with untreated cells.

## Discussion

Extracts from medicinal plants, particularly ethnomedicinal plants and their phytoconstituents, are reported to have anti-herpetic activity in cell cultures [34, 35]. The ethnomedicinal plant *P*. *tithymaloides* is used in Indian traditional medicine and has beneficial activities against skin ailments and microbial infections [14, 17]. To validate this use we have evaluated the antiviral activity of *P*. *tithymaloides* leaf extract and isolated compounds, along with their probable mode and mechanism of action against HSV-2.

HSV is one of the most common pathogenic viruses that inflicts a range of pathologies–from mild discomforts to lethal effects. Currently employed anti-herpetic drugs include the modified nucleotide analogue acyclovir and related prodrugs that inhibit viral thymidine kinase and/or DNA polymerase [36]. Although these drugs effectively reduce viral growth and multiplication, they fail to eliminate the virus from the infected individual, thereby increasing the risk of emergence of drug-resistant strains [37]. Therefore, new anti-HSV compounds, particularly non-nucleoside compounds and agents with different targets than the DNA polymerase are needed.

HSV infection is influenced by cellular signaling pathways and transcription factors, including activation of MRPK that promotes viral replication [38, 39]. Furthermore, HSV activates IκB kinase that triggers IκB-degradation in the early stage of infection resulting in a remarkable and unrelenting activation of NF-κB in epithelial, neuronal and lymphoid cells [40–42]. Supporting previous reports that secondary metabolites of plants that can inhibit NF-κB or MAPK activation also inhibit HSV infection [23], we found that MEPT and luteolin can inhibit replication of the clinical isolates and TK-deficient strains of HSV-2, accompanied with NF-κB effects. On contrast with the previous reports that HSV-2 activates MAPK [5, 33], we found neither MEPT nor luteolin affects HSV-2-induced MAPK and JNK activation.

The transcriptional regulator NF-κB plays a vital role in cell proliferation and viral gene expression of a number of viruses. Its activation prevents apoptosis of the host cell, which is essential for viral replication. It has been shown that the A and J type cyclopentemone prostaglandins (cyPG) are required for blocking TNF-α-induced NF-κB activation, through direct inhibition and modification of the IKKβ subunit of IKK [40], an effect related to the potent antiviral activity of cyPG. Curcumin, an inhibitor of NF-κB activation [43], has also been shown to inhibit HSV immediate early gene expression and replication [11]. However, resveratrol, a natural stilbenoid, suppresses HSV-induced NF-κB activation, which results in reduced expression of HSV genes and viral DNA synthesis [10, 24]. These findings concur with our results and reveal that compounds targeting signaling pathways may exert selective activity against a varied group of viral pathogens [44, 45].

HSV infection elicits production of various chemokines and cytokines, including TNF-α, IL-1β, IL-6, RANTES, and IFN-γ [46, 47]. Here, we observed that HSV infection of peritoneal macrophage leads to significantly increased production of TNF-α, IL-1β, IFN-γ and increased expression of iNOS. In contrast, MEPT- or luteolin-treated groups of animals exhibited significantly down-regulated production of TNF-α, IL-1β, IL-6, IFN-γ and iNOS expression.

## Conclusion

We have evaluated the *in vitro* antiviral activity of extracts of MEPT leaves and the isolated compound luteolin, as well as possible modes of action against HSV-2 and virus-induced inflammation. MEPT and luteolin exhibited strong antiviral activity (EC_50_) against all tested isolates of HSV-2 at 48.5–52.6 and 22.4–27.5 μg/m, respectively, far below their CC_50_ concentration (436.5 and 278.6 μg/ml); complete inhibition (EC_99_) of HSV-2 multiplication was achieved at 86.5 and 40.2 μg/ml, respectively, within 2–4 h post-infection. This was due to inhibition of NF-κB activation, but did not affect HSV-2-induced JNK and MAPK activation.

## Supporting Information

S1 File Extraction and isolation of luteolin and tetradecanediol, sodium salt from methanol extract of *P*. *tithymaloides* leaves. **Table A**, Assessment of cytotoxicity and ant-HSV activity of Tetradecanediol.
(DOC)
