# Supplementary material for: Pedilanthus tithymaloides Inhibits HSV Infection by Modulating NF-κB Signaling
Source: PLoS One. 2015 Sep 25;10(9):e0139338. doi: 10.1371/journal.pone.0139338 (PMC4583282; doi:10.1371/journal.pone.0139338)
Supplement: S1 File — Table A, Assessment of cytotoxicity and ant-HSV activity of Tetradecanediol. (DOC) [file pone.0139338.s001.doc]

**S1 File**

**Extraction and isolation**

The leaves of PT were thoroughly washed in running tap water, shade dried and powdered in a mechanical grinder. About 2 Kg of powdered leaves were defatted at room temperature using petroleum ether (3 x 4L), and then extracted successively using chloroform (3 x 4L) and methanol (3 x 4L) for 72h by cold maceration to obtain extracts of petroleum ether (150 g), chloroform (105 g) and methanol (85 g). These extracts when then tested for anti-HSV activity and we found that only the methanol extract (ME) had potent anti-HSV activity. Thus, ME was subjected to bioactivity guided fractionation. For isolation of possible compounds we used silica gel (60-120 mesh) column chromatography (CC), partitioned with n-Butanol and water saturated with n-Butanol (as n-Butanol is more polar than methanol, so most organic molecules will dissolved in Butanol). The aqueous part, before discarding, was further extracted with n-Butanol to ensure that no organic molecules remain in the aqueous extract. The n-Butanol-soluble fraction (46 g) was treated with Silica gel CC using a gradient of petroleum ether (100:0) to petroleum ether (60-80ºC)-CHCl_3_ (25:75), CHCl_3_ (100:0) to CHCl_3_-MeOH (70:30) and we obtained five major fractions. Out of these five fractions, studied with thin layer chromatography (TLC), only fractions 4 and 5 were active, and thus subjected to further study. TLC was used to in every step to identify all the fractions using β-sitosterol or β-sitosterol glucoside as marker compounds. Thus, the fraction 4 and 5, eluted with CHCl_3_-MeOH (100:0-90:10), was assembled and re-chromatographed on silica gel (100-200 mesh) CC using Petroleum ether-CHCl_3_ 50:50 to CHCl_3_-MeOH 85:15, to yield seven sub-fractions. Out of seven sub-fractions 4.1 to 4.4 showed the presence of β-sitosterol, while other three (4.5-4.7) was other than β-sitosterol. Thus, sub fraction 4.5-4.7 was added and further purified on SiO_2_ CC (CHCl_3_-MeOH 100:0 to 90:10) to afford **compound-1** (30 mg; yellow solid. m.p. 330-332^0^C; m/z 309). Similarly, fraction 5 eluted with CHCl_3_-MeOH (80:20-65:35) were grouped and submitted to silica gel CC (petroleum ether-CHCl_3_ 50:50 to CHCl_3_-MeOH 75:25) to get ten subfractions, of which subfractions 5.1 to 5.4 showed the presence of β-sitosterol glucoside and the rest was other than β-sitosterol glucoside. Thus, sub-fraction 5.5 to 5.8 was combined together and further purified on SiO_2_ CC (CHCl_3_-MeOH 95:5 to 85:15) to afford **compound-2** as white crystals (35mg; colourless solid, m/z 355.1535). The structures of the isolated compounds were determined by the spectral analysis of IR, NMR, and HR-ESIMS. The other fractions (petroleum ether and chloroform) mainly contain mixture of straight chain compounds, β-sitosterol and β-sitosterol glucosides (methanol fraction) showed no significant activity, and hence not used in further study.

**General procedures**

Melting points measured on a Yanagimoto Micro melting point apparatus are uncorrected. IR spectra were recorded on JASCO7300 FTIR spectrometer. ^1^H and ^13^C NMR spectra were recorded at 600 MHz and 150 MHz, respectively, using Bruker AVANCE 600 spectrometer with TMS as internal standard in C_5_D_5_N and or MeOD. ESI-MS and HR-ESI-MS were performed on a Q-TOF-micromass spectrometer. Silica gel (60 mesh, Merck, Germany) was used for CC, while TLC was carried out on silica gel 60F 254 (Merck, Germany) and spots were visualized by spraying Liebermann–Burchard reagent followed by heating. Preparative TLC was carried out on pre-coated silica gel l60 plates (thickness: 0.5mm; E. Merck, Germany). All other chemicals and solvents were purchased from SRL, Mumbai, India.

**Table A.** Assessment of cytotoxicity and ant-HSV activity of Tetradecanediol.

| **Virus** | **Tetradecanediol** | | | **Acyclovir** | | |
| --- | --- | --- | --- | --- | --- | --- |
|  | **CC_50_^a^** | **EC_50_^b^** | **SI^c^** | **CC_50_^a^** | **EC_50_^b^** | **SI^c^** |
| HSV-2G | 146.4 ± 6.33 | - | - | 128.8 ± 3.44 | 2.6 ± 0.8 | 49.53 |
| HSV-2 CI 1 | 146.4 ± 6.33 | - | - | 128.8 ± 3.44 | 2.8 ± 0.3 | 46.00 |

CI, Clinical Isolates 1;

^a^ CC_50_, 50% cytotoxic concentration for Vero cells in µg/ml;

^b^EC_50_, Concentration (µg/ml) producing 50% inhibition of virus-induced plaques in three separate experiments; ^c^SI, Selectivity index (CC_50_/EC_50_).
